# Neuroleukin/Autocrine Motility Factor Receptor Pathway Promotes Proliferation of Articular Chondrocytes through Activation of AKT and Smad2/3

**DOI:** 10.1038/srep15101

**Published:** 2015-10-13

**Authors:** Kang Tian, Weiliang Zhong, Xifu Zheng, Jinrui Zhang, Pixu Liu, Weiguo Zhang, Han Liu

**Affiliations:** 1 Department of Orthopaedics, First Affiliated Hospital, Institute of Cancer Stem Cell, Dalian Medical University, Dalian 116044, China

## Abstract

Cartilage defect is an intractable clinical problem. Therapeutic strategies for cartilage repair are far from optimal due to poor proliferation capacity of chondrocytes. Autologous chondrocyte implantation is a cell based therapy that uses *in vitro* amplified healthy chondrocytes from the patient. However, chondrocyte dedifferentiation during *in vitro* culture limits its application. Neuroleukin (NLK) is a multifunctional protein that stimulates cell growth and migration, together with its receptor autocrine motility factor receptor (AMFR, also called gp78). We investigated expression of NLK and AMFR/gp78 during cartilage development *in vivo* and in cultured articular chondrocytes *in vitro*, and found the pair associates with chondrocyte proliferation and differentiation. While applied to isolated articular chondrocytes, NLK promotes cell proliferation and secretion of type II collagen, a marker of proliferating chondrocytes. Further work demonstrates that NLK up regulates pAKT and pSmad2/3, but down regulates pSmad1/5. In animals, NLK treatment also promotes chondrocyte proliferation while inhibits terminal differentiation, leading to expanded proliferating zone but decreased prehypertrophic and hypertrophic zones in the growth plate region. NLK is therefore a candidate factor that can be applied in the treatment of cartilage defects.

Articular cartilage injury or loss is common in the department of orthopaedics, while therapeutic outcome is not always satisfactory, mainly due to poor self-regeneration of articular chondrocytes^1^. Since even minor cartilage defects may ultimately lead to tissue degeneration and onset of osteoarthritis, a better therapeutic strategy will greatly improve life quality of patients.

Chondrocyte is the sole cell type found in healthy articular cartilage, embedded in cartilaginous extracellular matrix composed of Collagen and Aggrecan^2^. Autologous chondrocyte implantation (ACI) is a cell-based therapeutic strategy to treat cartilage injuries (>2 cm^2^) without subchondral bone damage^3^, in which chondrocytes from healthy, non-bearing region of patient are cultured *in vitro* and then implanted in the lesion^4^. ACI has several advantages compared to alternative strategy such as microfracture, which relies on chondrocytes differentiated from mesenchymal stem cells released from microfracture sites. Nonetheless, treatment for cartilage defects like ACI also faces some challenges, among which formation of non-functional fibrocartilage resulted from chondrocyte dedifferentiation during *in vitro* expansion is most disappointing^5^,^6^,^7^. Hence, a successful method of *in vitro* chondrocyte amplification requires condition that maintains both proliferative and proper differentiated state of chondrocytes. We have previously looked at different culture conditions to amplify articular chondrocytes *in vitro*^8^,^9^. In this study, we sought to identify novel physiological relevant factors which can be used in expansion of articular chondrocytes.

Neuroleukin (NLK), also known as autocrine motility factor (AMF) and glucose-6-phosphate isomerase (GPI), is a multifunctional protein^10^. It is a neurotrophic factor for spinal and sensory neurons, a growth factor found in mouse salivary gland^11^,^12^, and an autocrine factor of cancer cells which binds autocrine motility factor receptor (AMFR) to enhance migration^13^,^14^,^15^. In the cell, GPI catalyzes isomerization between glucose-6-phosphate and fructose-6-phosphate in glycolysis and gluconeogenesis pathways. As a secreted factor, NLK not only stimulates metastatic and anti-apoptotic activities of tumor cells, also affects growth and migration of normal cells^16^,^17^,^18^. Many functions of NLK are initiated through its binding to AMFR/gp78, a 78 kDa transmembrane glycoprotein that was identified as autocrine motility factor receptor (AMFR) located on the cell surface, which also exhibits ubiquitin E3 ligase activity in the endoplasmic reticulum^19^,^20^. Both NLK and AMFR/gp78 are widely distributed including in osseous tissues. NLK has been reported to participate in osteoblast differentiation^21^,^22^. Interestingly, NLK expression is evident in proliferating chondrocytes, but not hypertrophic chondrocytes which are terminally differentiated^21^. However, the exact role of NLK/AMFR on chondrocyte proliferation and phenotype still remains unclear. In this study, we report that NLK/AMFR is implicated in chondrocyte proliferation and cartilage development.

## Results

### Expression of NLK and AMFR/gp78 in rat articular chondrocytes

**Figure 1:**
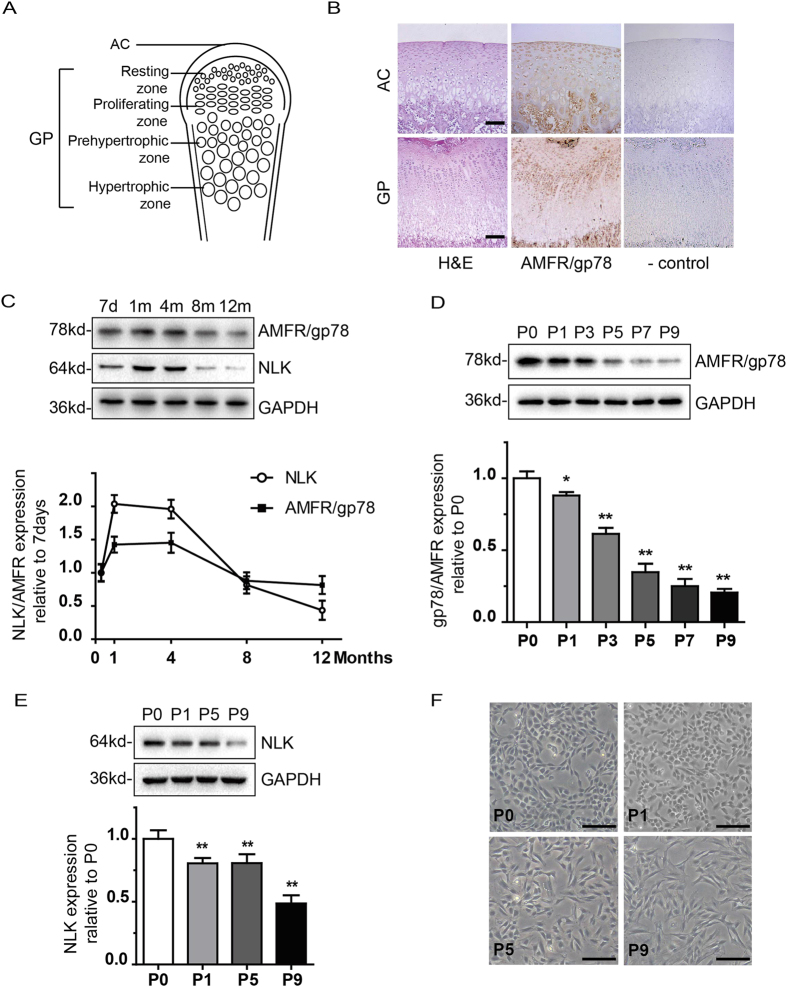
Expression of NLK and AMFR/gp78 in articular chondrocytes. (**A**) schematic diagram of femur head shows articular cartilage (AC) and growth plate (GP) composed of four layers. (**B**) immunohistochemistry of femur head from 1 month old rat stained with H&E, AMFR/gp78, and negative control, scale bar = 200 μm. (**C**) cartilage samples from knee joints of 7 days, 1, 4, 8, and 12 months old rats were homogenized, and proteins were analyzed with immunoblottings using antibodies indicated (GAPDH as loading control). Graph below shows quantification data from 5 independent experiments. (**D**–**F**), primary articular chondrocytes were isolated from 1 month old rat and cultured *in vitro*. AMFR/gp78 (**D**) and NLK (**E**) expression was examined in cells lysates from different passage numbers (P0-P9) as indicated. Lower panels show quantification data, n = 5. F shows change of cell morphology during *in vitro* passaging, scale bar = 200 μm. All error bars represent standard deviation, *(p < 0.05) and **(p < 0.01).

Firstly, we examined AMFR/gp78 expression in cartilage and growth plate region of 1 month old Sprague-Dawley rat. In cartilage of femoral head, AMFR/gp78 expression is apparent (Fig. 1A,B). While in growth plate region, AMFR/gp78 is more enriched in proliferating zone, also evident in resting zone, but absent in prehypertrophic and hypertrophic zones. These results are consistent with NLK expression reported previously^21^. Next, we compared expression of NLK and AMFR/gp78 in cartilage from rats of different age. In doing this, cartilage tissues from knee joints of Sprague-Dawley rats (7 days, 1, 4, 8, and 12 months old) were taken. Expression of NLK and AMFR/gp78 was examined by immunoblottings. As shown in Fig. 1C, levels of NLK and AMFR/gp78 appears to be correlated, which peaks at around 1 month (1.9 and 1.4 folds compared to 7 days respectively) and remains high until 4 months when animal already matured (when growth plate disappears and articular cartilage remains in the articular surface permanently), followed by rapid decrease (0.4 and 0.8 fold by 12 months respectively) with aging. Therefore, expression of NLK and AMFR/gp78 seems to be associated with cartilage development. We then looked at expression of NLK and AMFR/gp78 in cultured primary chondrocytes *in vitro*. Interestingly, levels of NLK and AMFR/gp78 drops quickly following passaging, with concomitant change of cell morphology from small polygonal to elongated spindle shape that indicates dedifferentiation of primary chondrocytes (Fig. 1D–F).

### NLK promotes proliferation of articular chondrocytes

**Figure 2:**
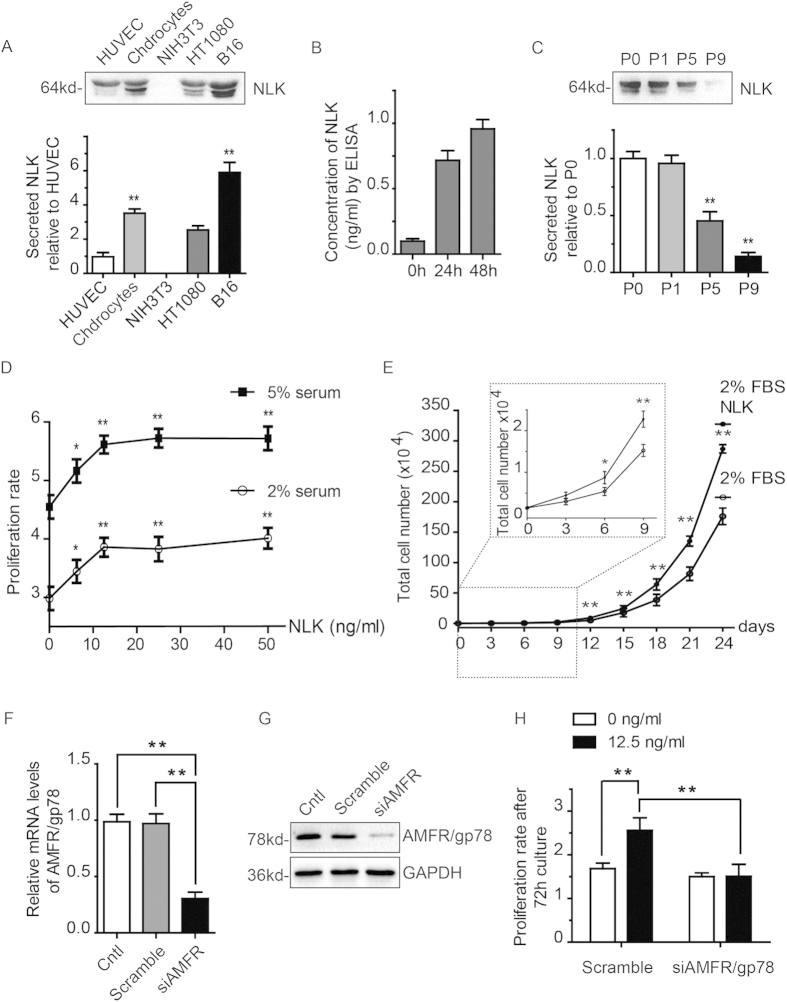
NLK promotes chondrocyte proliferation as a secreted factor. (**A**) cultured HUVEC, articular chondrocytes (AC), NIH3T3, HT1080, B16 cells were starved with serum free media for 24 hours, before secreted proteins were collected and analyzed by immunoblottings with NLK antibody. Lower panel shows quantification data, n = 5. (**B**) isolated chondrocytes were treated as in A for 24 and 48 hours. Media were collected and NLK secreted was determined with ELISA assay as described in the method section. (**C**) chondrocytes cultured *in vitro* were treated and analyzed as in A. (**D**) NLK was supplemented into 2% or 5% serum containing media at various concentrations (0, 6.25, 12.5, 25, and 50 ng/ml) that were used to culture primary articular chondrocytes over a 7 days period. Media were replenished every 2 days. Cell proliferation was then measured with MTT assays. Experiments were repeated 5 times, and proliferation rate was calculated and plotted. Each NLK treated group was compared to untreated control. (**E**) articular chondrocytes were cultured with or without 12.5 ng/ml NLK in 2% FBS media. Cells were harvested and counted at indicated time points using a cell counter (Countess® II FL, Life technologies). Data were summarized from experiments with chondrocytes isolated from 4 rats. (**F**,**G**) chondrocytes were treated with scramble or AMFR targeted siRNA for 3 days, before knockdown efficiency was measured with RT PCR and immunoblottings. (**H**) chondrocytes depleted of AMFR were stimulated with NLK for 3 days in the presence of AMFR or scramble siRNAs. Cell growth was measured with MTT assays and plotted as proliferation rates over 72 h. All error bars represent standard deviation, * and **(p < 0.05 and 0.01 respectively).

As mentioned above, a major role of NLK is to act as a secreted factor to regulate cell growth and migration. Furthermore, several growth factors such as TGFβ and BMP have been described to regulate chondrocyte proliferation in an autocrine/paracrine manner. Hence, we wonder whether NLK exerts its function on chondrocyte by similar means. We first examined whether chondrocytes secrete NLK. Isolated primary articular chondrocytes from knee joint cartilage of rat were starved for 24 hours before culture medium was collected. Secreted proteins were then precipitated with trichloroacetic acid and analyzed by immunoblottings with NLK antibody. HT1080 (fibrosarcoma), B16 (mouse melanoma), HUVEC (endothelial), and NIH3T3 (mouse fibroblast) cells were treated in parallel as controls. As shown in Fig. 2A, NLK secretion is evident for articular chondrocytes, HUVEC, HT1080, and B16, but not NIH3T3 cells. Furthermore, we determined concentration of secreted NLK from isolated chondrocytes using commercially available ELISA kit. Following 24 and 48 hours starvation, NLK secreted was at 0.72 and 0.95 ng/ml respectively (Fig. 2B). Considering above mentioned NLK decrease during *in vitro* expansion, we then examined NLK secretion from cultured articular chondrocytes of different passages. As expected, NLK secretion by chondrocytes also shows a decreasing trend following passaging (Fig. 2C).

Having identified correlated expression of secreted NLK and AMFR/gp78 in rat articular chondrocytes, we performed 3-(4, 5-Dimethylthiazol-2-yl)-2, 5-diphenyltetrazolium bromide (MTT) assays to examine how NLK affects growth of articular chondrocytes. Considering the results that expression of NLK and AMFR/gp78 in rat articular chondrocytes peaks at about 1 month and their levels drops quickly following passaging *in vitro*, unless specified, we performed all experiments with chondrocytes isolated from articular cartilage of 1 month old rats with no more than 1 passage. In MTT assay, we first supplemented various concentrations of NLK (0, 6.25, 12.5, 25, 50 ng/ml) in growth media containing either 2 or 5 percent fetal bovine serum (FBS). We measured cell proliferation with MTT method over a 7 days period (Fig. 2D). A similar trend is observed for both 2% and 5% FBS groups, in which NLK promotes chondrocyte proliferation in a dose depend manner that plateaus at around 12.5 ng/ml (Fig. 2D). As isolated chondrocytes are normally cultured for a rather long period in ACI to expand cells, we assessed the potential of NLK to stimulate chondrocyte proliferation over a 24 days period. As shown in Fig. 2E, NLK at 12.5 ng/ml continuously promotes chondrocyte growth over this long culture time. To assess involvements of AMFR/gp78 in NLK stimulated cell growth, we performed RNAi experiments to knock down AMFR in chondrocytes and repeated cell proliferation assays with NLK. Due to the fact that siRNA does not last long in the cell, we inspected influence of NLK on the growth of chondrocytes with AMFR/gp78 knockdown over a 3 days period following efficient depletion of AMFR/gp78 as revealed by RT PCR and immunoblotting results (Fig. 2F,G). As shown in Fig. 2H, NLK treatment leads to a significant increase of proliferation in chondrocytes treated with scramble siRNA (proliferation rates of 2.55 versus 1.68), while this promoting effect is abolished by AMFR/gp78 knockdown (proliferation rates of 1.52 versus 1.49). These findings suggest that AMFR/gp78 is required for NLK stimulated cell propagation.

**Figure 3:**
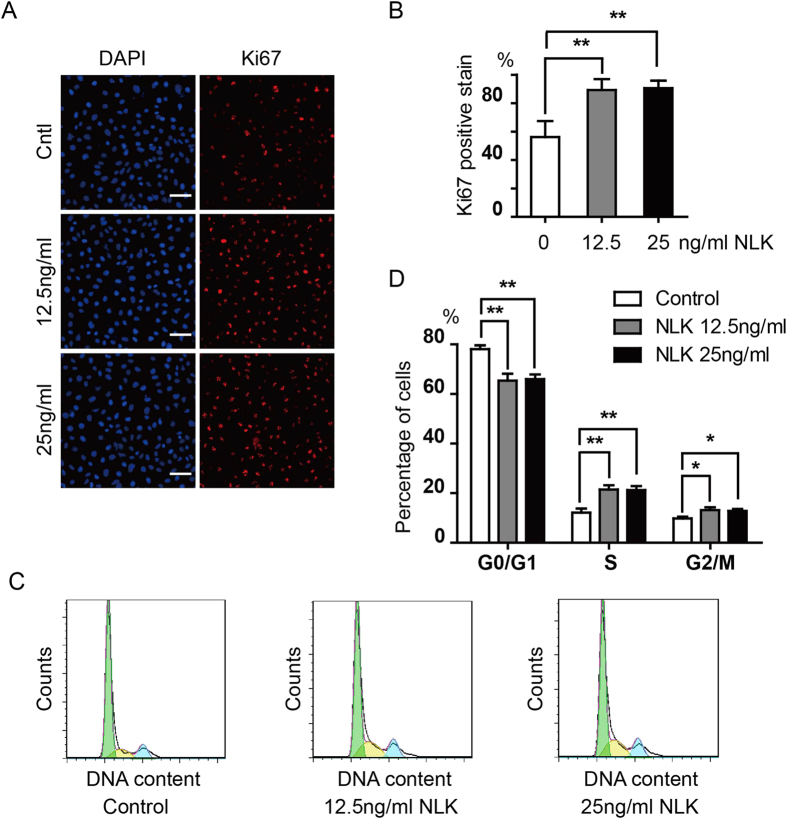
NLK increases proportion of proliferative chondrocytes. (**A**) cells cultured in NLK (12.5 and 25 ng/ml) or untreated as control (both with 2% FBS) were seeded onto coverslips before fixed and stained with DAPI and proliferation marker Ki67. (**B**) more than 300 cells were counted from 3 random views from A and percentages for Ki67 positive were calculated. Experiments were repeated 5 times. (**C**,**D**) cells were treated with NLK or untreated as control and stained with propidium iodine, prior to flow cytometry analysis. Data were analyzed by FlowJo and proportions for G0/G1, S, and G2/M were calculated. Representative histogram from 5 independent experiments is shown in (**C**) and quantification data are illustrated in (**D**). All experiments were carried out with 2% FBS containing medium. All error bars represent standard deviation, *(p < 0.05) and **(p < 0.01).

Moreover, we stained chondrocytes cultured in NLK supplemented media with Ki67, a proliferation marker. About 89 percent of cells treated with NLK (12.5 and 25 ng/ml) show positive stain, compared to around 55 percent for cells without NLK (Fig. 3A,B). Further, we carried out flow cytometry experiments to look at cell cycle distribution of NLK treated cells. Addition of 12.5 and 25 ng/ml NLK leads to comparable increase in proportion of cells in S and G2/M phases, from 19% to 34% in total (Fig. 3C,D). Overall, these findings suggest NLK promotes proliferation of articular chondrocytes *in vitro*.

### NLK takes part in phenotype maintenance of chondrocytes

**Figure 4:**
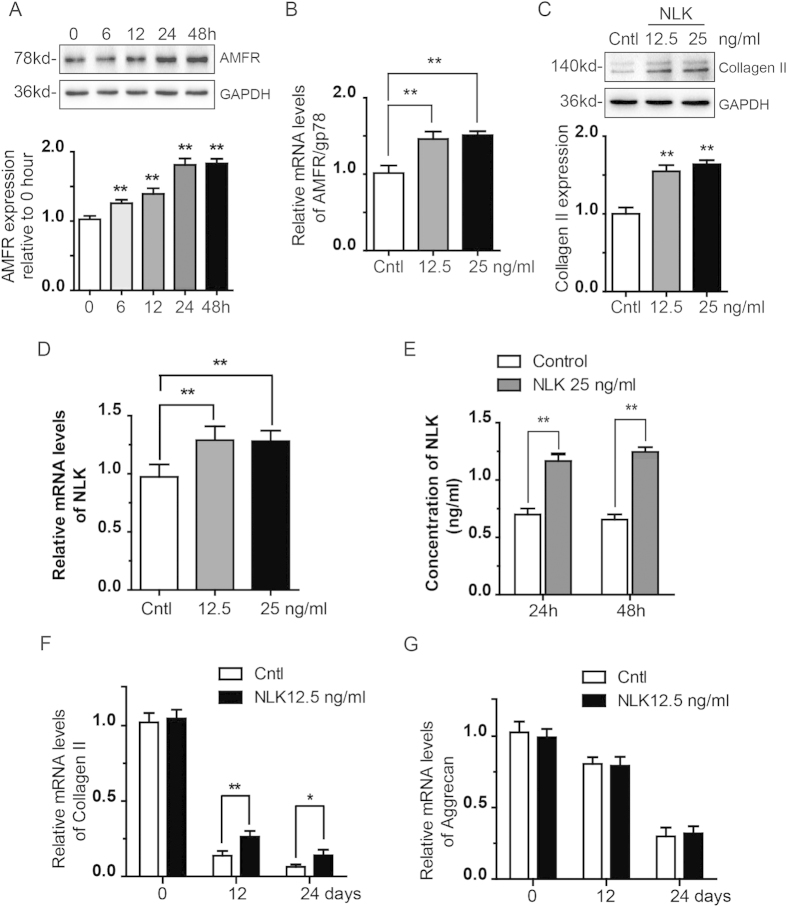
NLK induces expression of AMFR/gp78 and Collagen II. (**A**) isolated primary chondrocytes were treated with NLK at 25 ng/ml for 6, 12, 24, and 48 hours or left untreated as control before lysis. Lysates were analyzed by immunoblottings with AMFR/gp78 and GAPDH (loading control) antibodies. Lower panel shows data from 5 independent experiments. (**B**) RNA was extracted from cells treated with 12.5 and 25 ng/ml NLK for 48 hours and untreated ones as control. cDNA was made and quantitative PCR was performed to compare mRNA levels of AMFR/gp78. (**C**) cells were treated as in B and lysates were analyzed by immunoblottings with antibodies indicated. Lower panel shows quantification from 5 independent experiments. (**D**) cells were treated as in (**B**) and mRNA levels of NLK was measured by RT PCR. (**E**) NLK treated chondrocytes were starved following serial washes before secreted NLK was determined with ELISA. (**F**,**G**) quantitative PCR was performed to compare mRNA levels of Collagen II and Aggrecan following 12 and 24 days NLK treatment. All experiments were carried out with 2% FBS containing medium. All error bars represent standard deviation, and *(p < 0.05) and **(p < 0.01).

During *in vitro* expansion of chondrocytes in ACI, dedifferentiation tends to happen that results in formation of nonfunctional fibrocartilage. Therefore, optimal growth conditions that keep healthy state of chondrocytes are vital to success of ACI. Since AMFR/gp78 levels is associated with chondrocyte phenotype (Fig. 1D), we looked at effect of NLK on AMFR/gp78 expression. Interestingly, NLK addition up regulates AMFR/gp78 levels over a two days period, leading to a 1.7 folds increase by 48 hours (Fig. 4A). We then examined levels of AMFR/gp78 mRNA following NLK treatment for 48 hours, and found 1.49 folds increase compared to untreated (Fig. 4B). In addition, we assessed expression of Collagen II after NLK stimulation, which is considered as a specific marker of proliferating chondrocyte^23^. Using immunoblotting, we observed a 1.7 times upregulation of Collagen II by NLK (both 12.5 and 25 ng/ml) (Fig. 4C). We next wonder whether exogenous NLK can stimulate expression of endogenous NLK. As revealed by results from RT PCR and ELISA experiments, chondrocytes treated with exogenous NLK contain 1.35 folds more mRNA for NLK and secret considerably more amounts of NLK as compared to control treated cells, suggesting a positive feedback regulation that benefits phenotype maintenance of chondrocytes (Fig. 4D,E). Considering the potentials of NLK in ACI, we examined the effect of NLK on the expression of Collagen II and Aggrecan over long culture periods. Consistent with previous results, mRNA levels for both Collagen II and Aggrecan decrease with *in vitro* culture, even in the presence of NLK. However, chondrocytes treated with NLK contain 1.89 and 2.05 folds more Collagen II mRNA at day 12 and 24 respectively as compared to control cells, while mRNA levels of Aggrecan is not significantly altered by NLK treatment (Fig. 4F,G). Taken together, these findings suggest that chondrocytes cultured in NLK supplemented media are more prone to proliferation, rather than dedifferentiation.

### NLK regulation of signaling molecules in chondrocytes

**Figure 5:**
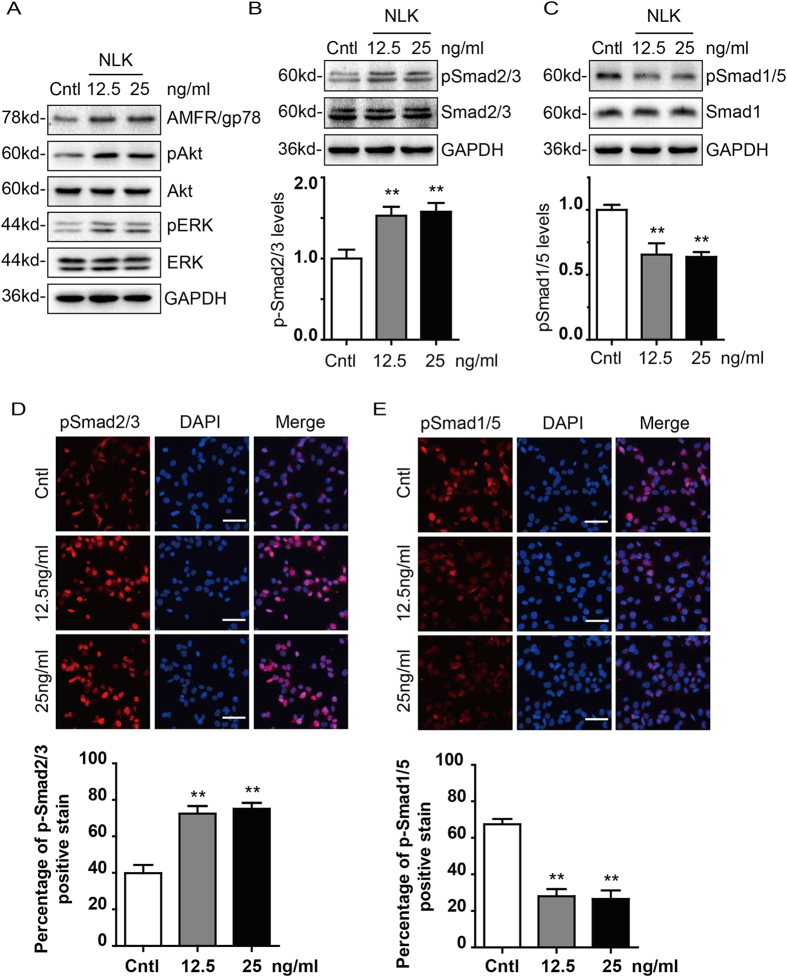
NLK up regulates phosphorylation of ERK1/2, AKT, and Smad2/3. (**A**) articular chondrocytes were treated with NLK (12.5 and 25 ng/ml) or left untreated for 48 hours before lysis. Lysates were analyzed by immunoblottings with antibodies indicated. (**B**,**C**) cells were treated as in A and lysates were probed for phosphorylated and total Smad2/3 (**B**) or phosphorylated Smad1/5 and total Smad1 (**C**). GAPDH is used as loading control. Lower panels show quantification from 5 independent experiments. (**D**,**E**) chondrocytes were seeded onto coverslips and then either untreated or treated with NLK (12.5 and 25 ng/ml) for 48 hours, prior to fixation and stain with pSmad2/3 and pSmad1/5. Scale bar = 50 μm. Lower panels show quantification data. All experiments were carried out with 2% FBS containing medium. All error bars represent standard deviation, and **(p < 0.01).

Having identified expression and roles of NLK in chondrocytes, we aim to understand the molecular mechanism by which NLK regulates chondrocytes. NLK and its receptor AMFR/gp78 have been reported to activate ERK1/2 and AKT in a number of cancer cells^24^,^25^,^26^. We thus carried out immunoblotting experiments to inspect levels of pERK1/2 and pAKT in NLK treated chondrocytes. As shown in Fig. 5A, both pERK1/2 and pAKT are up regulated following NLK stimulation, while total ERK1/2 and AKT remain unchanged. As expected, it seems in chondrocytes NLK may regulate ERK1/2 and AKT in a manner similar to tumor cells. Moreover, it is well established that Smad proteins play fundamental roles in proliferation and differentiation of chondrocytes. Activation of Smad2/3 and Smad1/5/8 by phosphorylation is associated with proliferation and terminal differentiation of chondrocytes respectively. To assess effect of NLK on Smad proteins, we first examined levels of pSmad2/3 and pSmad1/5 in NLK treated chondrocytes with immunoblottings, together with total Smad2/3 and Smad1. Compared to untreated cells, NLK treatment leads to increase of pSmad2/3 (to 1.6 fold) and concomitant decrease of pSmad1/5 signals (to 0.68 fold), but leaving total Smad2/3 and Smad1 constant (Fig. 5B,C). Additionally, consistent with immunoblotting data, immunofluorescence experiments illustrate that NLK treatment leads to elevated pSmad2/3 but reduced pSmad1/5 stains in the nuclei of chondrocytes (Fig. 5D,E). Collectively, these data suggest that NLK can stimulate multiple signaling pathways in chondrocytes to promote proliferation and prevent hypertrophy.

### NLK promotes chondrocyte proliferation *in vivo*

**Figure 6:**
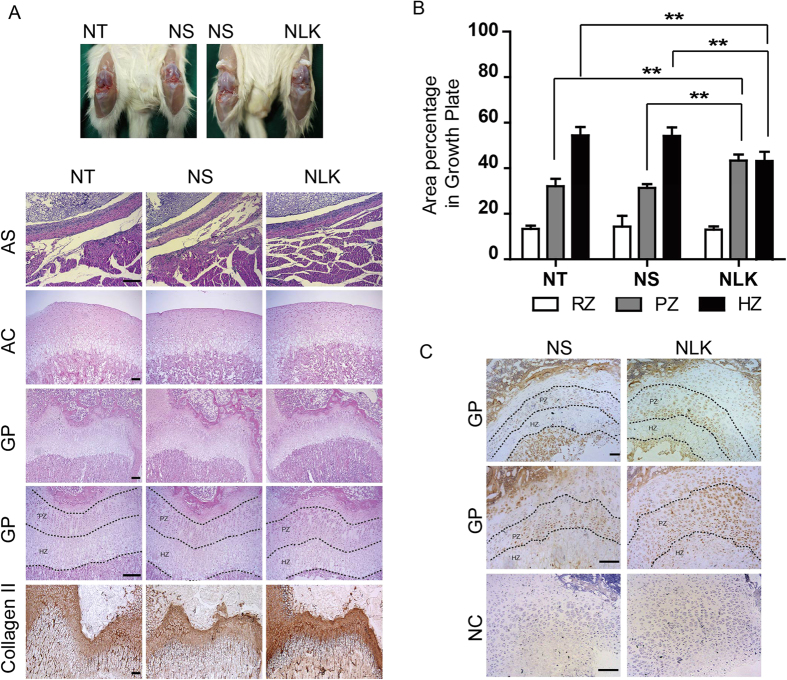
NLK expands proliferating zone of growth plate *in vivo*. (**A**) NLK (20 μl at 25 ng/ml) was injected into one knee joint of 5 days old rats (a group of 8 rats) every 3 days, while normal saline (NS) was injected into the other side as control. Another 3 rats were injected with NS into one knee joints while the other one with no treatment (NT). Animals were sacrificed at 30 days old. General inspection with gonarthrotomy observes no cartilage and bone defect from injected samples (top panel). H&E stains of articular synovial (AS) and articular cartilage (AC) appears normal for injected samples. Growth plate (GP) sections were stained with H&E as well as Collagen II, a marker for proliferating zone (PZ). NLK injected group shows expansion in proliferating zone, and correspondingly reduction in prehypertrophic and hypertrophic zones (together labeled as HZ), as well as elevated Collagen II staining, scale bar = 200 μm. (**B**) area of PZ, HZ, and total GP was calculated using ImageProPlus from NT, NS, and NLK samples (3, 8, and 8 respectively). Area percentage of RZ, PZ and HZ within GP was calculated and plotted. (**C**) tissue sections from growth plates of NS and NLK treated rat were stained with NLK. PZ and HZ were labeled, scale bar = 100 μm. All error bars represent standard deviation, and **(p < 0.01).

To evaluate the effect of NLK on cartilage growth and development *in vivo*, we set up animal experiments with newborn rats. NLK (20 μl at 25 ng/ml in normal saline) was injected into one knee joint, while normal saline was injected into the contralateral one of the same rat (5 days old) as control. The procedures were repeated every 3 days until 30 days (day 5–30) before animals were sacrificed. By general inspection, we observe no destruction of articular cartilage or bone erosion in injected animals (Fig. 6A). We examined synovial fluid with smear microscopy and observed no pathological changes from saline and NLK treated samples, compared to untreated group (data not shown). Moreover, H&E stain of synovium shows no sign of joint space exudate or inflammation in saline or NLK treated rats. We next inspected condyles of femur with H&E stains, which showed no morphometric difference between untreated and injected groups at articular cartilage region (Fig. 6A). Nonetheless, we observed significant changes at growth plate regions of NLK injected samples compared to untreated (NT) and saline injected (NS) ones. The proliferating zones from NLK treated group expand, leading to corresponding reduction of proportion of prehypertrophic and hypertrophic zones in the same growth plate region. We quantified these by calculating the area percentage of each zone in growth plate, and found proliferating zones in NLK treated group increased to 43% of growth plate, compared to ~26% in control samples. Correspondingly, total area percentage of prehypertrophic and hypertrophic zones in NLK treated group decreased to 43%, compared to ~57% in two control groups (Fig. 6B). Synthesis of type II collagen was also increased in NLK group as revealed by immunohistochemistry analysis (Fig. 6A). Similarly, NLK staining in the proliferating zone of growth plates in NLK injected rats is significantly stronger than that in the saline treated group (Fig. 6C). These *in vivo* data provide further evidence that NLK/AMFR functions to promote proliferation while inhibit terminal differentiation of chondrocytes.

## Discussion

The recent progress of tissue engineering has started to provide alternative strategies in the treatment of bone and cartilage defects, in combination with synthetic substitutes and bioactive factors to prepare functional replacement of bone and articular cartilage^27^,^28^. Clinical approaches adopted in the treatment of cartilage defects can be classified based on defect size and subchondral bone status^3^. These include marrow simulation based technique, osteochondral transplantation, and cell based technique, which are represented by microfracture, allograft/autograft, and ACI respectively. Microfracture requires a long postoperative rehabilitation period due to surgical injury^29^. Additionally, bone marrow mesenchymal stem cells (BMSCs) released from microfracture sites have the potential to proceed to terminal differentiation, leading to hypertrophic chondrocytes and endochondral ossification^30^. Patients treated with allograft/autograft based osteochondral transplantation need to undergo more surgeries, in addition to risk of donor-site morbidity in the long term^31^. In comparison, ACI is a two-step procedure relies on *in vitro* expansion of healthy autologous chondrocytes from biopsy. In ACI, cells are normally cultured for 4–6 weeks before re-implantation in the defect^3^. Nonetheless, chondrocyte dedifferentiation tends to happen during *in vitro* expansion and post-implantation, which may result in fibrocartilage formation rather than true cartilage and thus lead to significant deterioration in the long run^32^. Therefore, an optimized growth condition that maintains proliferative phenotype of chondrocytes will greatly improve ACI. Bearing this in mind, we focus our efforts on identifying novel physiological factors that associate with chondrocyte proliferation.

A number of growth factors, such as BMP, IGF1, and TGFβ, play fundamental roles in osseous tissues during both development and adult homeostasis, among which IGF1 and TGFβ are implicated in chondrocyte development and phenotype maintenance^33^,^34^,^35^. In this study, we identify NLK as another chondrocyte secreted factor that closely associates with chondrocyte proliferation and development, together with its receptor AMFR/gp78. NLK expression is previously reported for superficial articular chondrocytes, proliferating chondrocytes, and osteoblasts, but absent for hypertrophic chondrocytes^21^. Considering that NLK exerts many functions with its receptor AMFR/gp78, we investigated AMFR/gp78 expression in articular cartilage and growth plate regions of femoral head from 1 month old rat. In concert with NLK expression, AMFR/gp78 expression associates with proliferating chondrocytes but not terminal differentiated hypertrophic chondrocytes (Fig. 1). Moreover, we observed a similar pattern of protein levels change for NLK and AMFR/gp78 during rat development and aging. During *in vitro* passaging of isolated articular chondrocytes, a concurrent decreasing trend of NLK and AMFR/gp78 is obvious, with concomitant change of cell morphology due to dedifferentiation (Fig. 1). These results prompt us to speculate that NLK and AMFR/gp78 may cooperatively regulate cartilage development and chondrocyte proliferation. Although it should be noted that in immunoblotting experiments total cellular NLK and AMFR/gp78 were detected, which represent the collected pool of these molecules with various activities.

As a secreted factor, NLK regulates both normal and cancer cells^36^,^37^,^38^. In this study, we illustrate that articular chondrocytes secrete NLK which promotes their proliferation in an autocrine/paracrine manner (Fig. 2). As NLK from rat and human is highly conserved (~90% identity), we used recombinant human NLK proteins in this work. NLK treatment increased proportion of chondrocytes in S and G2/M phases of cell cycle and with positive Ki67 staining, leading to stimulated proliferation of isolated articular chondrocytes *in vitro* (Figs 2 and 3). Meanwhile, addition of NLK also elevated expression of AMFR/gp78 and Collagen II, two proteins associated with proliferative chondrocytes, in addition to NLK itself (Fig. 4). Conceivably, NLK/AMFR activates certain downstream signaling pathway and thus promotes chondrocyte proliferation in a feed forward way. More importantly, NLK is capable of promoting chondrocyte growth as well as Collagen II synthesis over long *in vitro* culture periods that are required for ACI.

Since NLK and its receptor AMFR/gp78 have been reported to activate PI3K-AKT and ERK/MAPK pathways in various cell lines^39^,^40^. We examined activation of AKT and ERK1/2 by immunoblottings in NLK treated cells, and observed a strong upregulation of pAKT as well as a moderate increase of pERK1/2. PI3K-AKT pathway is implicated in chondrocyte differentiation and endochondral bone growth during cartilage development^41^,^42^. Activation of AKT is observed in resting and proliferative chondrocytes but reduced during terminal differentiation. AKT activation enhances chondrocytes proliferation and survival, as well as synthesis of matrix proteins (proteoglycan and type II collagen)^33^,^43^,^44^. On the other hand, MAPK/ERK pathway is also involved in chondrocyte development, regulating multiple processes during cartilage formation, including chondrocyte proliferation and cartilage matrix remodeling^45^,^46^. Activation of ERK1/2 is prerequisite for insulin induced cell proliferation and differentiation in the model chondrogenic cell line ATDC5, as well as for chondrocyte proliferation induced by lysophosphatidic acid and mechanical compression^45^. Nevertheless, sustained activation of ERK1/2 shows inhibitory effects on chondrocyte proliferation and synthesis of matrix proteins, which can be reversed by MEK inhibitors^47^. Therefore, different amplitude and duration of ERK activation can lead to complete opposite outcome in chondrocyte proliferation and differentiation. In our experiments, NLK treatment resulted in strong stimulation of pAKT but only minor increase in pERK1/2, which might be more beneficial to chondrocyte proliferation.

TGFβ and BMP play pivotal roles during development of osseous tissues, signaling primarily through Smad proteins. Previous studies have confirmed the importance of balance between Smad2/3 and Smad1/5/8 activation in chondrocyte proliferation and differentiation^35^,^48^,^49^. In cartilage of young mice, TGFβ promotes proliferation of chondrocytes and deposition of cartilage specific extracellular matrix molecules (Aggrecan and Collagen II) via smad2/3 activation. While in cartilage of aging mice and experimental osteoarthritis models, activation of Smad1/5/8 triggers chondrocytes to undergo terminal differentiation, leading to chondrocyte hypertrophy and expression of terminal differentiation markers, such as type X collagen and MMP13^50^,^51^. Based on these, we examined the effect of NLK on activation of Smad2/3 and Smad1/5. As shown in Fig. 5, immunoblotting and immunofluorescence data demonstrate that NLK treatment results in elevated activation of Smad2/3 but decreased phosphorylation of Smad1/5, suggesting a proliferating status. So it seems that NLK/AMFR exerts their function on chondrocyte through multiple pathways, which function synergistically to promote proliferation. Despite the canonical role of TGFβ and BMP in regulating Smad signaling, Smad pathway can also be modulated by other upstream regulators like MAPK^52^,^53^,^54^. It is thus uncertain at this stage whether NLK stimulates Smad2/3 directly or indirectly through other signaling pathways.

In order to investigate the role of NLK in cartilage development, we performed *in vivo* experiments with newborn rats, which were treated with articular injection of NLK in one knee joint and normal saline in the other as control. As described in the results section, NLK treatment promotes chondrocyte proliferation while inhibits terminal differentiation, leading to expanded proliferating zone but reduced prehypertrophic and hypertrophic zones in the growth plate region (Fig. 6). Correspondingly, immunohistochemistry experiments with Collagen II, a specific marker for proliferating chondrocytes, show considerably stronger staining in NLK treated samples than those treated with normal saline or untreated, in parallel with increased accumulation of NLK (Fig. 6). These results are in harmony with data from BMP4 transgenic mice, which showed opposite observation of decreased proliferating zone and increased hypertrophic zone resulting from activation of Smad1/5/8.

On a separate note, NLK/GPI disposition has been reported in joints of rheumatoid arthritis. Kouskoff *et al.* proposed that GPI serves as an auto-antigen for B and T cells in K/BxN T cell receptor (TCR) transgenic rheumatoid arthritis (RA) mice model^55^. Since immune cells are continuously exposed to NLK/GPI under physiological condition, autoimmune response may be caused by loss of tolerance. Although the exact role of GPI and anti-GPI immunoglobulin G in RA still remains a subject of controversy, as both high and low correlation was reported previously by researchers^56^,^57^. The intra-joint application of NLK/GPI needs to be rigorously examined with caution. In our *in vivo* experiments, intra-joint injection of exogenous NLK did not cause pathological changes. One possible explanation may lie within the local concentration of NLK. We used at 25 ng/ml, while in joints of rheumatoid arthritis much higher concentration (>4000 ng/ml) was reported^58^.

We herein report association of NLK/GPI and AMFR/gp78 with cartilage development *in vivo* and chondrocyte proliferation *in vitro*. Cells are normally cultured for 4 to 6 weeks before re-implantation in the lesion during ACI, thus further investigation is required to test the effect of NLK on human chondrocytes over this long period^3^. In addition, because NLK only partially inhibits chondrocytes dedifferentiation in 2D culture, we also seek to extensively explore applications of NLK in various settings, such as in 3D culture, under hypoxia condition, and in combination with other bioactive factors. NLK is therefore a potential candidate factor in ACI to assist in both *in vitro* expansion of chondrocytes and *in vivo* cartilage repair after implantation, which may also provide benefits for other therapeutic strategies in the treatment of cartilage defects.

## Materials and Methods

All experiments were performed in accordance with the 1996 National Institutes of Health Guide for the Care and use of Laboratory Animals, and the experimental procedures were approved by the Institutional Animal Care and Use Committee of Dalian Medical University.

### Antibodies and other reagents

The following antibodies were used: rabbit anti-Ki67 (Abcam, UK); rabbit anti-NLK and mouse anti-AMFR (Santa Cruz Biotechnology, CA); mouse anti-GAPDH (ProteinTech Group, USA); rabbit anti-Akt, rabbit anti-phospho-Akt (Ser473), rabbit anti-ERK1/2, mouse anti-phospho-ERK1/2, rabbit anti-Smad2/3, rabbit anti-phospho-Smad2/3, mouse anti-Smad1, and rabbit anti-phospho-Smad1/5 (Cell Signaling Technologies, USA); rabbit anti-Collagen type II antibody (Boster, China). Human recombinant Neuroleukin/Glucose 6 phosphate isomerase full length protein was obtained from Abcam (UK).

### Chondrocyte isolation and cell culture

Articular cartilage was obtained from femoral heads and condyles of 1 month old Sprague-Dawley rats, weighing 80–120 g. Cartilage was separated from the subchondral bone and cut into small pieces using a sterile surgical blade in sterile PBS. Primary chondrocytes were isolated by digestion with 0.2% type II collagenase (Gibco, USA) for 4 hours at 37 °C in a shaking water bath and resuspended in Dulbecco’s modified Eagle’s medium/F-12 (Hyclone, USA) containing 10% fetal bovine serum (Gibco, USA). The experimental protocols were approved by the Institutional Animal Care and Use Committee of Dalian Medical University. Cells were cultured in a humidified incubator at 37 °C in 5% CO_2_. Cell lines used in this study: human HT-1080 fibrosarcoma and murine B16 melanoma cells (Keygen biotech), human umbilical vein endothelial cells (HUVEC) and murine embryonic fibroblast NIH-3T3 cells (ATCC, USA). Cells were maintained in Dulbecco’s modified Eagle’s medium (Gibco, USA) supplemented with 10% fetal bovine serum (Gibco, USA) at 37 °C with 5% CO_2_.

### Cell proliferation assay and RNAi

Cell proliferation was measured with 3-(4, 5-dimethylthiazol-2-yl)-2, 5-diphenyltetrazolium bromide tetrazolium (MTT) (Sigma) assay. Briefly, primary articular chondrocytes were seeded at a density of 2000 cells/well into 96-well-plate in growth medium. After initial cell seeding, cells were serum starved before treated with various concentrations of NLK (6.25, 12.5, 25, and 50 ng/ml) in growth media containing 2 or 5% FBS. Medium was replenished every other day. At indicated time, MTT was added and cells were placed back into incubator for 3 hours, before DMSO addition and absorbance reading at 570 nm. siRNA oligos were obtained from GenePharma (Shanghai, China). The sequences are as follow: AMFR, 5′-GCAACAUCUGGUUAUCUAUTT-3′ and 5′-AUAGAUAACCAGAUGUUGCTT-3′. A scramble siRNA oligo was used as negative control. siRNA was introduced into cells using Lipofectamine 2000 (Life technologies) reagent according to manufacturer’s instructions.

### Enzyme-linked immunosorbent assay (ELISA)

Isolated chondrocytes were cultured in 10 cm dishes until about 90% confluence, before starvation with serum free medium for 24 and 48 hours. Cells were checked under microscope to make sure no apparent cell death happened. Medium was collected and spun down to remove any particulates. Samples were concentrated using an Amicon Ultra centrifugal filter (Millipore) with a molecular weight cutoff of 10,000 Da to a final volume of 500 μl, from which NLK concentration was determined using a rat NLK/GPI ELISA kit (Lengton) according to manufacturer’s instructions.

### Cell cycle analysis

Cell cycle was analyzed by flow cytometry using PI (propidium iodide) (Sigma) staining. Briefly, chondrocytes were starved for 12 hours before NLK treatment for 48 hours in growth media containing 2% FBS. Cells were harvested, and 1 million cells were fixed in ice cold 70% ethanol for 12 hours, prior to staining in 50 μg/ml propidium iodide. Samples were analyzed on a FACScan flow cytometer (BD, USA) and data analyzed with FlowJo software.

### Cell lysis and immunoblottings

Cells were lysed on ice in RIPA lysis buffer (10 mM Tris-HCl pH7.5, 150 mM NaCl, 1% w/v Triton X-100, 0.1% w/v SDS, 1% sodium deoxycholate) supplemented with mammalian protease inhibitors and phosphatase inhibitor mixture (Sigma). Equal amounts of protein were separated by SDS-PAGE, and transferred to nitrocellulose blotting membrane (Merck Millipore, USA). Membranes were blocked with 5% nonfat milk in Tris-buffered saline with 0.1% Tween20 (TBST), incubated with indicated primary antibodies with gentle shaking at 4 °C overnight. After incubation with HRP coupled secondary antibodies (Thermo Fisher Scientific, USA) for 1 hour at room temperature, membranes were treated with enhanced chemiluminescence (ECL) reagent (Advansta, USA) and developed using ChemiDoc MP imaging system (Biorad, USA).

### TCA precipitation

Trichloroacetic acid (TCA) precipitation assay was used in this study to harvest secreted proteins from cultured cells in conditioned medium. Briefly, 4 × 10^6^ cells were seeded into 10 cm dish and cultured in growth medium containing 10% FBS. Next day, cells were starved with medium without FBS. After 24 and 48 hours, medium was collected and treated with 10% ice cold TCA. Samples were mixed by vortexing before centrifugation for 10 minutes at 14,000 × g. Pellet was washed with 200 μl acetone and centrifuged again. Samples were dried completely before dissolved in SDS sample buffer, followed by immunoblotting analysis.

### Immunohistochemistry

After anesthesia by lidocaine injection, femoral heads and condyles were obtained from 1 month old Sprague-Dawley rats. Articular cartilage from each animal was cleared of soft tissue, and fixed in 4% paraformaldehyde, prior to decalcification in 10% EDTA (pH 7.4) at room temperature as descirbed^59^,^60^, and then embedded in paraffin for serial 5 μm section preparation. The experimental protocols were approved by the Institutional Animal Care and Use Committee of Dalian Medical University. For immunohistochemistry, all sections were deparaffinized using xylene and rehydrated with graded alcohol. Each section was blocked with blocking solution (Maxin, China) for 30 minutes at room temperature. Primary antibody was diluted with PBS and incubated overnight at 4 °C, and secondary antibody at room temperature for 1 hour. A DAB staining kit (Maxin, China) was used for DAB stain, and all procedures were followed according to manufacturer’s instructions. Finally sections were mounted and viewed using a Leica microscope.

### Immunofluorescence

Cells were seeded onto glass coverslips (0.8 × 0.8 cm) and cultured in 6 well plate until reaching 50% confluence. Coverslips were washed twice with PBS, fixed with 4% paraformaldehyde for 15 minutes, and blocked with 5% BSA in PBS for 1 hour at room temperature. Primary antibody was added for 1 hour. After incubation with fluorescent secondary antibodies (Invitrogen, USA) at room temperature for 1 hour, coverslips were mounted and examined under a fluorescent microscope (Leica, Germany).

### Real time PCR

Total RNA was extracted with Trizol Reagent (Invitrogen, USA). cDNA was generated using a PrimeScriptRT reagent Kit (TAKARA) according to manufacturer’s instructions. Real time PCR was performed using SYBR Select Master Mix (Applied Biosystems, USA). All reactions were performed in triplicates. Data were analyzed by normalization to an internal control gene, beta-Actin. The sequences of primers used were as follow: Aggrecan (forward 5′-GGCCTTCCCTCTGGATTTAG-3′ and reverse 5′-CCGCACTACTGTCCAAC-3′), Collagen II (forward 5′-CCCCTGCAGTACATGCGG-3′ and reverse 5′-CTCGACGTCATGCTGTCTCAAG-3′), AMFR/gp78 (forward 5′-TTCGCTACCTGTTCCATGAGG-3′ and reverse 5′-GGCTTCCATGTTTCCTACCAC-3′), NLK (forward 5′-TGAAGACGCCCCTGGATAAG-3′ and reverse 5′-CCATGTCACCCTGCTGGAA-3′), and beta-Actin (forward 5′-AGGGAAATCGTGCGTGAC-3′ and reverse 5′-CGCTCATTGCCGATAGTG-3′).

### Statistical analysis

All data presented were summarized from at least five independent biological experiments and were shown as mean ± standard deviation (SD). Statistical significance was determined using Student’s t test (two tailed) in GraphPad Prism software, p < 0.05 was considered significant.

## Additional Information

**How to cite this article**: Tian, K. *et al.* Neuroleukin/Autocrine Motility Factor Receptor Pathway Promotes Proliferation of Articular Chondrocytes through Activation of AKT and Smad2/3. *Sci. Rep.* **5**, 15101; doi: 10.1038/srep15101 (2015).
